# Humoral Immunity to Hantavirus Infection

**DOI:** 10.1128/mSphere.00482-20

**Published:** 2020-07-15

**Authors:** Taylor B. Engdahl, James E. Crowe

**Affiliations:** a Department of Pathology, Microbiology and Immunology, Vanderbilt University, Nashville, Tennessee, USA; b Vanderbilt Vaccine Center, Vanderbilt University Medical Center, Nashville, Tennessee, USA; c Department of Pediatrics, Vanderbilt University School of Medicine, Nashville, Tennessee, USA; University of Michigan-Ann Arbor

**Keywords:** B cell responses, antibody function, bunyavirus, hantavirus, neutralizing antibodies

## Abstract

Hantaviruses are zoonotic pathogens found in parts of Europe, Asia, South America, and North America, which can cause renal and respiratory failure with fatality rates up to 40%. There are currently no FDA-approved vaccines or therapeutics for hantavirus-related diseases; however, it is evident that a robust neutralizing antibody response is critical for protection from severe disease. Although virologists first described this family of viruses in the 1950s, there is limited information on the neutralizing epitopes that exist on the hantavirus antigenic glycoproteins, Gn and Gc, and sites important for the design of effective therapeutics and vaccines. We provide a thorough summary of the hantavirus field from an immunological perspective. In particular, we discuss our current structural knowledge of antigenic proteins Gn and Gc, identification of B cell neutralizing epitopes, previously isolated monoclonal antibodies and their cross-reactivity between different hantavirus strains, and current developments toward vaccines and therapeutics. We conclude with some outstanding questions in the field and emphasize the need for additional studies of the human antibody response to hantavirus infection.

**IMPORTANCE** Hantaviruses are pathogens that sometimes pass from animals to humans, and they are found in parts of Europe, Asia, and North and South America. When human infection occurs, these viruses can cause kidney or lung failure, and as many as 40% of infected people die. Currently, there are no vaccines or therapeutics for hantavirus-related diseases available. A first step in developing prevention measures is determining what type of immune response is protective. Increasingly it has become clear that the induction of a type of response called a neutralizing antibody response is critical for protection from severe disease. Although virologists first described this family of viruses in the 1950s, there is limited information on what features on the surface of hantaviruses are recognized by the immune system. Here, we review the current state of knowledge of this information, which is critical for the design of effective therapeutics and vaccines.

## Abstract (precis)

Hantaviruses are pathogens that sometimes pass from animals to humans, and they are found in parts of Europe, Asia, and North and South America. When human infection occurs, these viruses can cause kidney or lung failure, and as many as 40% of infected people die. Currently, there are no vaccines or therapeutics for hantavirus-related diseases available. A first step in developing prevention measures is determining what type of immune response is protective. Increasingly it has become clear that the induction of a type of response called a neutralizing antibody response is critical for protection from severe disease. Although virologists first described this family of viruses in the 1950s, there is limited information on what features on the surface of hantaviruses are recognized by the immune system. Here, we review the current state of knowledge of this information, which is critical for the design of effective therapeutics and vaccines.

## INTRODUCTION

Hantaviruses are members of the order *Bunyavirales* and are global emerging pathogens transmitted by rodents (1). Hantaviruses are endemic worldwide and categorized into two different groups based on geography and pathogenesis of infection. Old World hantaviruses, including Hantaan (HTNV), Puumala (PUUV), Seoul (SEOV), and Dobrava (DOBV), cause hemorrhagic fever with renal syndrome (HFRS) with a 1% to 15% mortality rate and 100,000 to 150,000 cases per year (2). New World hantaviruses, including Andes (ANDV) and Sin Nombre (SNV) viruses, cause hantavirus cardiopulmonary syndrome (HCPS) with a case fatality rate of 40% but are less frequent, with a few hundred cases a year (2). Hantaviruses spread through the inhalation of aerosolized rodent feces; however, studies of recent outbreaks of ANDV infection have reported human-to-human transmission (3). The National Institute of Allergy and Infectious Diseases (NIAID) has classified hantaviruses as category A pathogens, highlighting concerns of high mortality rates, ease of transmission, and lack of medical countermeasures.

There are currently no licensed vaccines or therapeutics for hantavirus infection; however, clinical trials have commenced using active immunization of experimental DNA vaccines or passive transfer of polyclonal immune serum (4). Additional studies have produced recombinant human monoclonal antibodies (MAbs) from survivors of ANDV infection and shown therapeutic efficacy in animal models (4–10). Finally, clinical research has shown that high neutralizing antibody titers correlate with increased survival in hantavirus infection (11). Thus, a robust humoral immune response to hantavirus infection is critical for surviving infection, but the molecular and structural basis for a protective human neutralizing antibody response is not well characterized for hantaviruses. This review will cover what we currently understand about the humoral immune response to hantavirus infection, specifically focusing on the neutralizing antibody response, and conclude by identifying the knowledge gaps that would aid in the rational design of vaccines and therapeutics.

## ANTIGENIC TARGETS OF HANTAVIRUS NEUTRALIZING ANTIBODIES

Hantaviruses are trisegmented, enveloped, negative-sense RNA viruses whose genomes encode four structural proteins (1). The medium (M) segment of the genome encodes the glycoprotein precursor, a conserved sequence that host proteases cleave to yield an N-terminal glycoprotein, Gn, and a C-terminal glycoprotein, Gc (12). Gn/Gc glycoproteins arrange into square-shaped spikes extending ∼10 nm from the lipid envelope, and there is no apparent organization of the spikes on the virion (13–16). Cryo-electron microscopy of hantavirus particles reveals pleomorphic morphologies, with average diameters ranging from 70 to 150 nm, with no symmetry in the arrangement of glycoprotein spikes on the viral envelope (14–18). Molecular weight analysis suggests that the spike is composed of four Gn protomers and four Gc protomers; however, the complex arrangement and interface of Gn/Gc on hantaviruses remains largely unknown (14, 15).

The N-terminally located glycoprotein, Gn, forms the distal portion of the spike and is solvent exposed (16, 19). The function of the Gn protein is currently unknown; however, it has been proposed to aid in the stabilization the prefusion Gn/Gc complex and in receptor binding and entry into cells (12).

Multiple host factors and potential receptors have been identified to facilitate hantaviral entry including integrins, decay-accelerating factor (DAF/CD55), gC1qR, and protocadherin-1 (PCDH-1) (20). β_3_ integrins have been shown to facilitate the entry of pathogenic hantavirus species causing HFRS and HCPS but impact entry of nonpathogenic species including Tula and Prospect Hill virus (PHV) (21, 22). Antibodies targeting α_V_β_3_ on human umbilical vascular endothelial cells (HUVECs) were able to decrease infectivity of pathogenic species (SNV, ANDV, HTNV, SEOV, PUUV, and NY-1V), while antibodies targeting α_5_β_1_ decrease infectivity of nonpathogenic PHV. Additionally, ANDV and HTNV neutralizing antibodies were shown to inhibit binding of endothelial cells to platelets, possibly indicating a role in mediating vascular permeability (23). Numerous integrins require recognition of a tripeptide arginine-glycine-aspartate (RGD) motif for cell adhesion; however, hantaviral glycoproteins lack an RGD motif, and direct interactions of hantavirus Gn/Gc proteins with integrins have not yet been shown (21, 22). *In vitro* studies also have shown that DAF/CD55 and gC1qR play a role in HTNV and PUUV infection (24, 25). Most recently, studies have suggested that the host protein PCDH-1 may be involved specifically in the entry of New World hantaviruses, including ANDV and SNV, and Gn/Gc proteins directly interact with the extracellular cadherin repeat 1 (EC1) domain of PCDH-1 (26). It is possible that Gn mediates binding to EC1 and entry, but the molecular determinants of hantaviral glycoprotein engagements are unknown. Antibodies targeting the EC1 domain of PCDH-1 show a titratable decrease in viral infectivity on HUVECs. The hantaviral entry as a target of neutralization is still being elucidated.

Sequence analysis of the Gn proteins in different hantaviruses has shown that Gn exhibits a higher frequency of mutation and may be under selective pressure by the humoral immune response (16). In contrast, Gc is less exposed on the glycoprotein spike, and the sequence of Gc shows greater conservation between different hantaviruses than Gn (16). Similar to many other enveloped viruses, hantaviruses require fusion of the viral and host cell membranes to deliver the genome to the cytoplasm of the cell to be transcribed (19, 27–30). Hantaviruses are taken up in the cell, and low pH in the endosome allows for conformational changes in the surface glycoproteins to induce fusion. Gc has a characteristic class II fusion protein fold consisting of three domains. In low pH, domain III makes a large conformational change revealing a hydrophobic fusion loop that then is inserted into the host cell membrane (27, 29). The postfusion form of Gc is a homotrimer that is able to fold back in on itself to bring the two membranes in close proximity and fuse the endosomal and the viral envelope together. Gn may shield the fusion loop on Gc from prematurely triggering and promoting fusion as described in other bunyaviruses, but this has not been shown in hantaviruses (16, 17). However, functional studies have shown a role for temperature in modulating fusogenic activity, indicating that dynamics in the Gn and Gc may uncover the fusion loop as the temperature increases (31). The incomplete knowledge of the structure of the glycoprotein spike has prevented a full understanding of how these proteins interact to perform essential roles in the viral entry and the fusion process. Additionally, the lack of complete structural information has made it challenging to discover potential sites of vulnerability on these proteins.

## PREVIOUSLY ISOLATED MAbs

There have been relatively few studies characterizing humoral immunity to hantavirus infection through the isolation of antibodies (Table 1) (11, 16, 32–36). For Old World hantaviruses, previous studies have isolated MAbs against Hantaan (HTNV) or Puumala (PUUV) viruses. Several groups isolated murine hybridoma-derived MAbs in the 1980s against HTNV (35, 37, 38). Antibodies isolated following viral challenge in these studies demonstrated that both Gn and Gc are targets of neutralizing antibodies. A follow-up study by Schmaljohn et al. with 15 anti-HTNV MAbs demonstrated that both Gn and Gc neutralizing MAbs could prevent productive HTNV infection in hamsters, while hamsters receiving passive transfer of nonneutralizing antibodies sustained productive infection (39). Four neutralizing MAbs also were isolated from a human survivor of HTNV infection using phage display library panning, and all MAbs showed specificity for Gc (40). Phage display techniques do not preserve the naturally occurring pairing of heavy and light chains, but the interaction of MAbs with viral proteins often is driven principally by heavy chain interactions. Lundkvist and Niklasson also generated two neutralizing MAbs from rodents (bank voles) after PUUV challenge, one targeting Gn (5A2) and one targeting Gc (4G2) (41). The researchers then used these MAbs in order to direct isolation of four anti-Gc human MAbs from a patient with idiopathic thrombocytopenia purpura and demonstrated that one MAb, designated 1C9, showed neutralizing activity against multiple PUUV strains (42). However, passive transfer of 1C9 did not protect hamsters from PUUV challenge (43). The MAbs described have shown neutralizing capacity and some therapeutic potential, but there are limited follow-up studies or new studies isolating Old World antibodies through improved technologies.

**TABLE 1:** Linear epitopes mapped to hantavirus glycoproteins from previously isolated monoclonal antibodies or human sera from convalescent survivors of infection

Species	Method	Target	MAb/serum	Neutralizing activity?	Residues	Amino acid sequence	Reference(s)
PUUV (strain Sotkamo)	Phage display peptide library	Gn	5A2	Yes	61–72, 264–280	SLKLESSCNFDL, EPLYVPTLDDYRSAEVL	33, 34
		Gc	1C9	Yes	822–834	EQTCKTVDSNDCL	
		Gc	4G2	Yes	904–921	KCAFATTPVCQFDGNTIS	
PUUV (strain Sotkamo)	Phage display peptide library	Gn	Human serum	Not tested	22–30	VNAKNLNEL	34
		Gn		Not tested	61–72	SLKLESSCNFDL	
		Gn		Not tested	82–96	FTKWTWETKGDLAEN	
		Gn		Not tested	442–453	TVYCNGVKKVIL	
		Gc		Not tested	946–966	SALEWIDLDSSLRDHINVIVS	
SNV (isolate 3H226)	Western blotting of truncated proteins	Gn	Human serum	Not tested	59–89	LKIESSCNFDLHVPATTTQKYNQVDWTKKSS	36
HTNV (strain 76-118)	Peptide scan	Gc	M7, Y1, Y5, Y7, Y22	Yes	916–924, 954–963	KVMATIDSF, LVTKDIDFD	40
ANDV (strain CHI-7913)	Peptide scan	Gn	Human serum	Yes	14–26	TLTLAMPKTTYEL	32
	Peptide scan	Gc		Yes	955–967	NLVLNRDVSFQDL	
	Peptide scan	Gc		Yes	691–703	RKLTNPANKEES	

For New World hantaviruses, only ANDV-neutralizing MAbs have been reported (44, 45). A 2018 study by Garrido et al. described the isolation of recombinant MAbs by antigen-specific B cell sorting of ANDV Gn/Gc-reactive B cells from human survivors of ANDV infection (45). Researchers identified two neutralizing MAbs that can protect Syrian hamsters after exposure; however, the mechanisms of neutralization by which these MAbs operate and their antigenic targets are unknown (45). A more recent paper described the isolation of 19 ANDV-specific mouse hybridoma-derived MAbs after challenge with a recombinant vesicular stomatitis virus (VSV)/ANDV Gn/Gc or plasmids encoding Gn/Gc from multiple hantavirus species (ANDV, HTNV, PUUV) and also demonstrated postexposure protection in Syrian hamsters (44). Twelve of these MAbs showed neutralizing activity against wild-type ANDV, and interestingly, most of the MAbs from the ANDV-only challenge reacted with Gn, while the MAbs from the challenge with multiple species reacted with Gc. Studies with previously isolated antibodies suggest key patterns of activity, including Gn and Gc reactivity, neutralizing activity, and protection in animals, but there is still a significant lack of knowledge of the human antibody response to hantaviruses, especially to New World virus species. New antibody isolation technologies have facilitated the generation of anti-ANDV MAbs, but epitopes and mechanisms of neutralization are still unknown.

## B CELL EPITOPES

**FIG 1:**
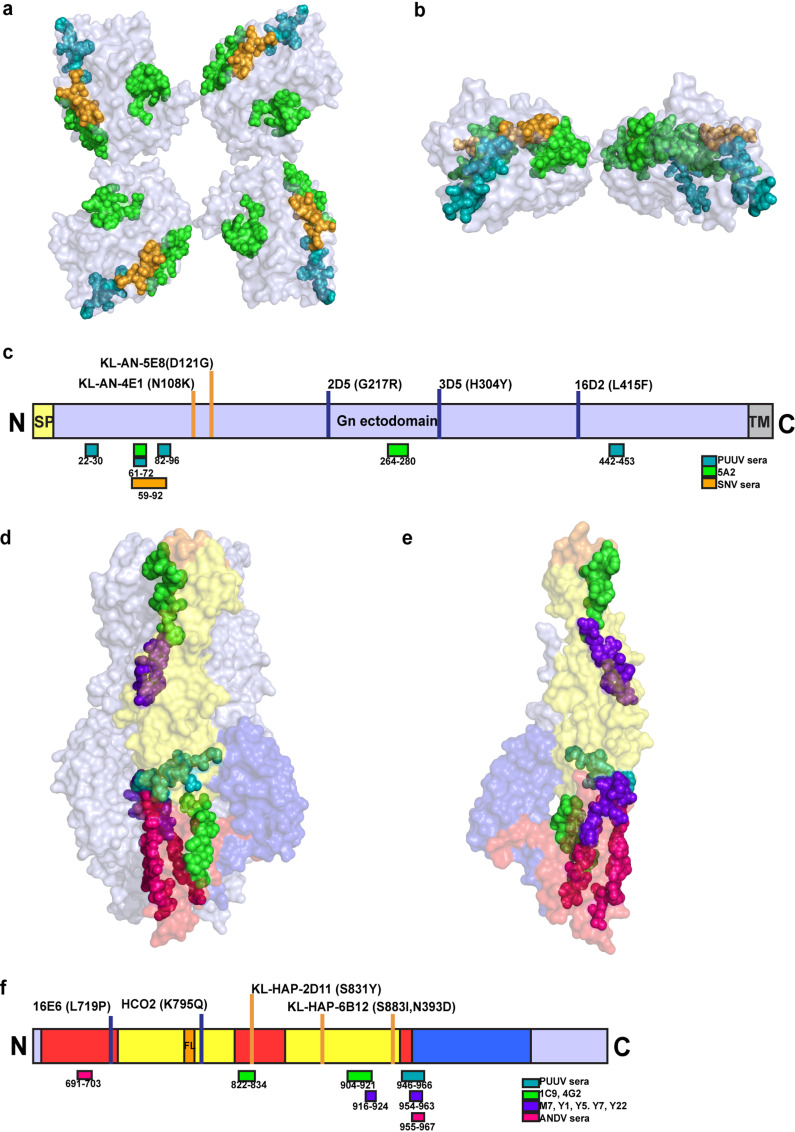
Previously identified antigenic sites on hantavirus glycoprotein Gn or Gc. (a and b) Neutralizing MAbs and epitopes for serum antibody recognition identified by peptide scanning and generation of escape mutant viruses are mapped on the crystal structure of Puumala virus Gn protein (PDB: 5FXU) in the tetrameric form on the surface of the virion. Gn is shown as a tetramer from the top view (a) or dimer from the side view (b). (c) Epitopes and escape mutations also are mapped to the linear genome of Gn. The signal peptide (SP) or transmembrane domain (TM) is highlighted in yellow or gray, respectively. (d and e) Previously identified neutralizing MAb and serological epitopes identified by peptide scanning and generation of escape mutant viruses are mapped on the crystal structure of Puumala virus Gc in the postfusion form of trimer (PDB: 5J9H). Gc is shown as a trimer (d) or protomer (e) to demonstrate epitopes that are inaccessible in the trimer. (f) Epitopes and escape mutations also are mapped to the linear genome of Gc. Domains I, II, and III are indicated in red, yellow, or blue, respectively, and the fusion loop is indicated in orange. Spheres indicating epitopes are color coded as follows: green, PUUV MAbs (5A2, 1C9, 4G2); purple, HTNV MAbs (M7, Y1, Y5, Y7, Y22); orange, sera from convalescent Sin Nombre patients; teal, sera from convalescent Puumala patients; pink, sera from convalescent ANDV patients. Escape mutations are indicated by the blue (anti-HTNV MAbs) or orange (anti-ANDV MAbs) bars.

Since there is a lack of knowledge of the structural and atomic level details of the Gn/Gc hetero-oligomer, there is also a lack of knowledge of important epitopes in the antibody response to hantaviruses. Currently, epitopes on the hantavirus glycoprotein spike have been identified only through linear peptide scanning (Table 1) or generation and sequence analysis of escape mutant viruses, and the field has not identified conformational epitopes through study of antigen-antibody complexes. Most of the MAb and serological epitopes previously identified on Gn map to the solvent-exposed region of the protein (Fig. 1a) (34, 36) Numerous reactive peptides for human sera or PUUV MAbs also overlap on the outer edge of the glycoprotein spike, which may also be more solvent exposed on the virion (Fig. 1b) (15, 16). Also, the changes in ANDV escape mutant viruses generated with mouse MAbs are located in epitopes found near the N-terminal region of the ectodomain, while the mutations allowing escape from anti-HTNV MAbs do not cluster in the same area (Fig. 1c). Interestingly, two of the three MAbs that mapped to Gn did not show a complete clearance of HTNV in animals; thus, these sites may not be fully protective (39).

Previously isolated neutralizing antibodies to PUUV and HTNV hantaviruses also map to epitopes located on Gc (Table 1) (33, 40, 46, 47). Mapping of these epitopes on Gc indicated that these antibodies may sterically hinder conformational changes needed during the fusion process, since some sites are not accessible in the postfusion trimeric form of Gc (Fig. 1d). Like other class II fusion proteins, Gc consists of three structural domains. Domain I at the N terminus links both domain II, which contains the highly conserved fusion loop, and domain III, which undergoes significant conformational change to form the postfusion trimer. Immunoprecipitation and structural studies have demonstrated that bank vole PUUV MAbs may recognize a B cell epitope on the Gn-Gc interface, and predicted epitopes are exposed in prefusion models of Gc but blocked in postfusion models by domain III (Fig. 1e) (13, 29, 41, 47). Other neutralizing antibodies, however, bind to domain II near the fusion loop, indicating that some antibodies may neutralize by steric hindrance of trimer formation during fusion (Fig. 1f) (29, 47). MAbs targeting domain I may inhibit the movement of domain III during fusion, while MAbs targeting domain II may block trimerization or insertion of the fusion loop into the host cell membrane (29). Antibodies in neutralizing sera from convalescent patients previously infected with PUUV and ANDV also map to similar sites on Gc, specifically domain II and the linker region of domain I that links to domain III (Fig. 1f) (32, 34). Escape mutant viruses generated by anti-HTNV and anti-ANDV MAbs also localize to domains I and II (44, 46). Gc also is less exposed on the glycoprotein spike compared to Gn, and the Gc sequence shows greater conservation between different hantaviruses than Gn (27, 29). This conservation could be due to the critical role of the protein in fusion and decreased pressure from humoral immunity. Thus, these neutralizing epitopes may point to conserved sites on the protein and show which antigenic sites on Gc are accessible by the antibodies (16). Epitopes have not been mapped to domain III on Gc, likely due to its proximity to the membrane and shielding from the humoral immune response by Gn. Recombinant ANDV domain III also was shown to inhibit cell-to-cell fusion and trimer formation; thus, targeting domain III with MAbs also may be an effective way to neutralize virus (48). Epitopes identified on Gn and Gc cluster in specific regions on the proteins, which may contribute to neutralizing activity or highlight immunodominant sites. Mapping the recognition sites for additional MAbs through structural studies, especially those from human survivors, will help us understand the important antigenic sites on the hantavirus glycoprotein spike.

## CROSS-REACTIVITY OF THE HANTAVIRUS IMMUNE RESPONSE

There are hundreds of hantavirus species endemic worldwide; however, only six species cause the majority of hantavirus-related diseases (2). Old World hantaviruses (HTNV, PUUV, SEOV, and DOBV) cause HFRS, or vascular leakage primarily targeting the kidneys. New World hantaviruses (SNV and ANDV) cause HCPS, also characterized by the same general type of vascular leakage pathology, but primarily targeting the lungs. The full-length M segment encoding Gn/Gc has ∼50% to 80% amino acid similarity between the six major pathogenic hantavirus species, which suggests that there may be highly conserved antigenic sites across Old World and New World species. Serological studies of HFRS patient sera following a single infection have demonstrated modest neutralizing activity for at least two species of hantaviruses, while most HCPS patient serum had neutralizing activity across Old World (HTNV, SEOV, PUUV, DOBV) and New World (SNV) hantaviruses (49, 50). Studies at the U.S. Army Medical Research Institute of Infectious Diseases (USAMRIID) also have evaluated cross-reactivity and cross-protection through the use of DNA vaccines bearing the M segment encoding Gn/Gc from different hantavirus species. Vaccination with the HTNV M segment protects against HTNV, SEOV, and DOBV in hamsters, but not PUUV or ANDV (7, 9). To create a pan-hantavirus vaccine, Hooper and colleagues designed a mixed vaccine containing HTNV/PUUV/ANDV/SNV M segments that elicited cross-neutralizing antibodies to all four species but failed to induce neutralizing antibodies to SEOV or DOBV (51). Previously isolated MAbs also showed a range in breadth across several virus species. Human anti-HTNV Gc-specific MAbs cross-neutralized HTNV, SEOV, and DOBV but not PUUV (40). Murine anti-HTNV Gc-specific MAbs also reacted with PUUV but only neutralized HTNV (37). Serologic, polyclonal, and monoclonal antibody studies suggest that there likely are broadly reactive and neutralizing antigenic sites on the glycoprotein spike, especially on Gc, but there is still a fundamental lack of knowledge of where these epitopes are positioned on the glycoproteins. It is also unclear to what extent epitopes recognized by cross-reactive MAbs can be used to design broadly protective therapeutics and vaccines.

## VACCINES, NEUTRALIZING ANTIBODIES, AND PROTECTION

Many studies have demonstrated the importance of a neutralizing antibody response in hantavirus disease severity and protection. Clinical studies testing patient sera for neutralizing antibodies have shown that a low neutralizing IgG titer is associated with moderate to severe disease outcomes in patients with HFRS and HCPS (11, 52, 53). Furthermore, high neutralizing antibody titers against SNV and ANDV persist years after initial infection (53). Compassionate use treatment involving the passive transfer of hyperimmune ANDV human sera to treat HCPS showed a decrease in the case fatality rate, but the efficacy could not be statistically evaluated (54).

Vaccination strategies to elicit neutralizing antibodies were investigated through a multitude of different platforms. In South Korea, Hantavax, a formalin-inactivated HTNV vaccine produced in the brain of suckling mice, is licensed for HFRS (55). However, with the current vaccination strategy of two doses, the neutralizing seroconversion rate was 23.2% after only 1 month postvaccination (56). A phase I and II study evaluated the safety and efficacy of a vaccinia virus vector-based HTNV vaccine, but the results showed a lack of substantial neutralizing antibody response just 6 months after administration (39, 57). Preclinical studies testing a recombinant vesicular stomatitis virus (VSV) vaccine bearing the ANDV glycoprotein genes showed complete protection in hamsters and elicited a neutralizing antibody response that was correlated with long-term protection from ANDV challenge (58, 59). Thus, inactivated or chimeric virus vaccines show potential, but we need more research to increase immunogenicity. It is possible that inactivation destroys key epitopes or that chimeric vaccines do not properly display neutralizing antigenic sites, thus affecting their immunogenicity.

The most promising current vaccine approach is the use of DNA vaccines containing the M segment, which includes the Gn and Gc genes, from HTNV (9), SEOV (60), PUUV (6), SNV (51), and ANDV (7). Hooper et al. have shown in phase I clinical trials that combination PUUV/HTNV DNA vaccines administered through intramuscular electroporation (IM-EP) were safe and elicited a long-lasting neutralizing antibody response, and additional clinical trials testing safety and efficacy of other iterations are under way (4, 5). Furthermore, ANDV DNA vaccination induces serum neutralizing antibodies in nonhuman primates (7), geese (61), ducks (62), and transchromosomal bovines (8) and can protect hamsters from ANDV challenge pre- and postexposure (8). Previous vaccination efforts highlight the importance of specifically targeting Gn and Gc to generate a robust and long-lasting neutralizing antibody response and to produce an effective treatment or prophylactic regimen for hantavirus infection.

## CONCLUSIONS AND OUTSTANDING QUESTIONS

Studies described in this review have highlighted the importance of humoral immunity, specifically neutralizing antibodies, in the treatment of HCPS and HFRS. Although there have been laudatory efforts in the structural studies of hantavirus glycoproteins, characterization of previously isolated antibodies, and vaccination efforts, understanding the humoral immunity to hantaviruses is only beginning. Studies with previously isolated MAbs begin to answer questions regarding protection, therapeutic efficacy, and neutralization potency, but there is still very limited knowledge of human MAbs generated from hantavirus infection. Although numerous linear epitopes and escape mutants have been characterized, there is still little information on conformational epitopes recognized by the humoral immune response. Understanding the ultrastructural arrangement and dynamics of Gn and Gc on the surface of the virion and how that may contribute to the exposure or occlusion of important antigenic sites is critical in development of medical countermeasures. For example, understanding the antigenic sites exposed in the respiratory syncytial virus (RSV) F protein and molecular determinants of prefusion stabilization has led to the development of a highly immunogenic RSV vaccine (63). Most recently, knowledge of the molecular-level dynamics of the receptor binding domain (RBD) in coronaviruses gave way to the rapid development of a prefusion stabilized spike protein vaccine that exposes immunogenic sites in the “up” form of the RBD (64).

Although some studies have indicated the existence of common antigenic sites shared by several hantavirus species, we have yet to identify neutralizing sites conserved on the hantavirus Gn and Gc proteins. The only way to combat the emergence of future novel hantaviruses is to have a clear understanding of critical, conserved epitopes shared by all hantaviruses. For human immunodeficiency virus (HIV), broadly neutralizing MAbs have led to the design of immunogens eliciting fusion peptide-directed responses with significant cross-clade breadth (65). Additionally, isolation of a broadly reactive influenza virus antibody identified a novel epitope in the hemagglutinin trimer interface that will contribute to universal influenza vaccine design (66).

The foundational work summarized in this review supports the importance of understanding more about the humoral immunity of hantaviruses, particularly the antigenic sites on Gn and Gc targeted by neutralizing antibodies elicited during hantavirus infection. Despite important progress in characterizing the hantavirus humoral immune response, further work must be done to understand the role of neutralizing antibodies in protection, and to rationally design vaccines and therapeutics.
